# Screening for acid sphingomyelinase deficiency in patients with an interstitial lung disease

**DOI:** 10.1186/s13023-026-04352-z

**Published:** 2026-05-12

**Authors:** Marie Vermant, Ilse Van Raemdonck, Nathalie Veyt, Marianne S. Carlon, Emanuela Elsa Cortesi, Nico De Crem, Marieke De Rooy, Tine Follet, Stefan Gogaert, Bart Vanaudenaerde, David Cassiman, Laurens J. De Sadeleer, Wim A. Wuyts

**Affiliations:** 1 Department of Chronic Diseases and Metabolism, KU Leuven, Leuven, Belgium; 2 Pulmonology Department, UZ Leuven, Leuven, Belgium

Acid Sphingomyelinase Deficiency (ASMD), also known as Niemann Pick disease (NPD) types A, A/B and B, is a rare, autosomal recessive lysosomal storage disorder [1]. There are three subtypes of ASMD, namely type A, A/B and B. Infantile neurovisceral ASMD (ASMD type A) typically presents at a young age, with a rapidly progressive phenotype. The median survival typically does not exceed the third year of life [1].

In addition to this infantile neurovisceral form, two chronic forms exist [1]. First, ASMD type A/B, or chronic neurovisceral ASMD, and second, ASMD type B or chronic visceral ASMD. An overview of the involved organs in both the chronic neurovisceral and chronic visceral subtypes can be found in Fig. 1. Typically, both subtypes present with hepatosplenomegaly, growth delay, dyslipidemia, thrombocytopenia, and interstitial lung disease. The main difference between the chronic neurovisceral and chronic visceral subtypes lies in the presence of neurological symptoms (e.g., learning difficulties, ataxia, developmental delays). ASMD type A/B is also associated with coarser facial features. These two phenotypes exhibit slower disease progression and a prolonged survival rate compared to the infantile neurovisceral form [2, 3].

**Fig. 1:**
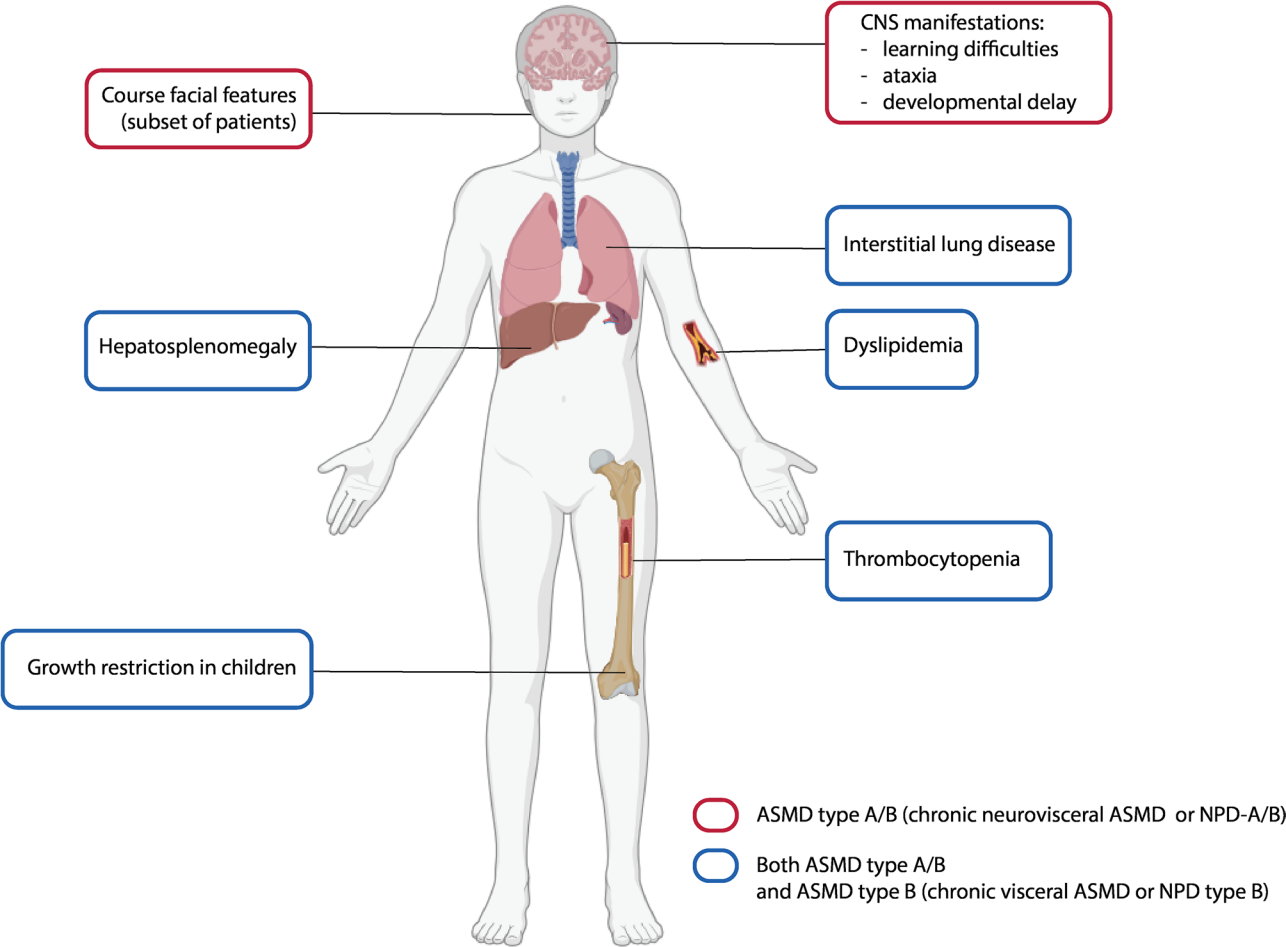
Figure an overview of the affected organs in acid sphingomyelinase deficiency types A/B and B

A prevalence of 0.53 cases per 100,000 live births has been reported in the Netherlands [4]. Initial diagnostic testing is performed by determining the acid sphingomyelinase enzyme activity via dried blood spot (DBS) [2]. Subsequently, the diagnosis is confirmed by genetic testing. ASMD is caused by pathogenic variants in the *SMPD1* gene. This leads to low or absent activity of acid sphingomyelinase and a subsequent lysosomal accumulation of sphingomyelin.

A known feature of ASMD types B and A/B is pulmonary involvement, which typically presents as interstitial lung disease (ILD). The term ILD encompasses a large, heterogeneous group of disorders. ILD primarily affects the interstitium [5], a thin, complex network of connective tissue that lies between the alveolar epithelium and the capillary endothelium, but can also occur by the accumulation of intra-alveolar substances [6]. The diagnosis of an ILD is complex, incorporating clinical history, pulmonary function testing, bronchoscopy, and radiology. When uncertainty remains, histopathological evaluation may be required. ILDs typically present with exertional dyspnea, dry cough, and, in advanced disease, respiratory failure and death.

Pulmonary involvement in ASMD is associated with a high mortality rate. In ASMD types A/B and B, respiratory failure is a primary cause of death, equaling the registered liver-related mortality [7]. A mortality rate of 44.4% due to respiratory complications was reported in symptomatic ASMD patients over 18 years of age [3]. In patients with ASMD, ILD is caused by an accumulation of lipid-laden macrophages in the lung tissue [8–10]. Typical CT-graphic findings on High-Resolution chest CT (HRCT) are the presence of interlobular septal thickening, ground-glass opacities, intralobular lines, and a crazy-paving pattern [11]. Despite these observations, data on ILD in ASMD remain remarkably scarce, particularly regarding its epidemiology, clinical presentation, and natural history.

Treatment of ASMD consists of enzyme replacement therapy, namely olipudase alfa, a recombinant human acid sphingomyelinase [12, 13]. In a phase 2/3 trial, olipudase alfa was well-tolerated, improved diffusion capacity of the lung and decreased hepatosplenomegaly. It is not indicated for the neurological manifestations of ASMD as it does not cross the blood/brain barrier.

This study aimed to assess the presence of an underlying ASMD in a population of patients with an interstitial lung disease.

Two-hundred and fifty adult patients with ILD were consecutively included for ASMD screening, in the ILD outpatient clinic. Exclusion criteria included the diagnosis of sarcoidosis. Informed consent was obtained upon inclusion (Ethics Committee University Hospitals Leuven, S67353). Baseline characteristics including sex, age, ILD working diagnosis, comorbidities, body weight, and pulmonary function parameters were collected. All patients underwent DBS testing. Five milliliters of blood were collected and pipetted onto the DBS card. The anonymized DBS card was subsequently sent to Archimed Life Science (Vienna, Austria) for enzyme activity assays. Abnormal or borderline results were followed by *SMPD1* genetic testing. Genetic testing was performed by next generation sequencing of all coding exons and flanking intronic regions after DNA extraction from the DBS card.

73% (182/250) of patients were male, 72% (180/250) were ever-smokers. Median FVC%pred was 83% (IQR 28) upon inclusion, median DLCO%pred was 48% (IQR 20). Table 1 shows the patient characteristics. 6.4% (16/250) of patients had abnormal or low-normal enzyme levels (acid sphingomyelinase (ASM) < 1.2 µmol/L/h) or increased Lyso-sphingomyelin (SPM) > 70 ng/ml, and therefore underwent genetic testing. One patient had a borderline increased SPM level (71.3) with a normal ASM level (1.3), 15 patients had decreased levels of ASM (range: 0.6–1.2 µmol/L/h) with normal SPM levels (range 30.3–64.5 ng/ml). No cases of ASMD were identified in this unselected ILD population. Three patients were heterozygous for a variant of unknown significance in the *SMPD1* gene. Both detected variants of unknown significance were two missense variants: c.1474G > A (p.(Gly492Ser)) in two patients, and c.872G > A (p.(Arg291His)) in one patient.

**Table 1:** Patients characteristics

		*n*	%
**Male**		182	73%
**Smoking Status**			
	Non Smoker	70	28%
	Ex Smoker	178	71%
	Current Smoker	2	1%
**ILD working diagnosis**			
	IPF	183	73%
	fHP	16	6%
	CPFE	14	6%
	uILD	12	5%
	CTD-ILD	9	4%
	iNSIP	5	2%
	PPFE	2	1%
	Other	9	4%
**Comorbidities**			
	Gastro-esophageal reflux	129	52%
	Hematological disease	19	8%
	Hepatological disease	33	13%
	Osteoporosis	34	14%
	Emphysema	75	30%
	Arterial Hypertension	132	53%
	Hypercholesterolemia	162	65%
	Diabetes Mellitus	58	23%
	Cardiac atherosclerosis	72	29%
	Chronic Renal Insufficiency	36	14%
		**Median**	**IQR**
**Age**		73.0	12
**BMI**		26.6	5.24
**FVC%pred**		83	28
**TLC%pred**		76	23
**DLCO%pred**		48	20

IPF= idiopathic pulmonary fibrosis, fHP= fibrotic hypersenstivity pneumonitis, CPFE= combined pulmonary fibrosis and emphysema, uILD=unclassifiable interstitial lung disease, CTD-ILD=connective tissue disease related interstitial lung disease, iNSIP= idiopathic non specific interstitial pneumonia, PPFE=Pleuroparenchymal fibroelastosis, %pred = percent predicted, FVC=functional vital capacity, TLC=total lung capacity, DLCO = gas transfer

In this cohort of 250 consecutively screened ILD patients, no cases of ASMD were detected. Our results align with the very low prevalence of ASMD reported in population-based studies. Given such rarity, a more targeted approach may be preferable. Previous reports suggest that ASMD should be considered when ILD occurs in combination with (hepato)splenomegaly, thrombocytopenia, dyslipidemia characterized by low HDL cholesterol, or unexplained liver disease [14]. Additionally, a decision tree algorithm has recently been developed to aid in identifying patients with a possible ASMD [14]. DBS testing offers a minimally invasive, practical first-line tool for identifying patients who require further genetic evaluation.

This study has several limitations. It was conducted in a single tertiary care center, and although the sample size was relatively large for a rare disease, it remains small compared with the true population prevalence. Additionally, the median age at inclusion is high for a lysosomal storage disease. Several patients demonstrated decreased ASM activity in the absence of pathogenic SMPD1 variants. While this is an intriguing finding, no definitive mechanistic explanation can currently be provided. Most reductions were borderline and may reflect biological variability or assay-related variation. This uncertainty should be considered when interpreting isolated reductions in ASM activity in ILD populations. Nevertheless, this study represents the first systematic DBS screening effort for ASMD in an unselected ILD population, providing valuable data on feasibility and diagnostic yield. Future multicentric studies should be organized to increase sample size and to improve diagnostic yield.

The availability of enzyme replacement therapy with olipudase alfa has significantly changed the clinical landscape. Whereas diagnosis previously had limited therapeutic consequences, recognition of ASMD is now critical, directly impacting patient management and prognosis. This study demonstrates the feasibility of large-scale DBS screening and provides a foundation for more targeted approaches that could ensure timely identification of patients likely to benefit from olipudase alfa treatment. The study also highlights a high false positive rate, which may complicate screening strategies, for example in high-risk populations, but also in newborn screening. Future strategies may be most effective when focused on ILD patients with additional ASMD features, such as (hepato)splenomegaly, thrombocytopenia, or dyslipidemia.

## Data Availability

All data is available upon reasonable request.
